# Targeting to BMP9 to restrain flare-up of fibrodysplasia ossificans progressiva

**DOI:** 10.1038/s44321-024-00180-5

**Published:** 2024-12-03

**Authors:** Qiwen Li, Quan Yuan

**Affiliations:** State Key Laboratory of Oral Diseases & National Center for Stomatology & National Clinical Research Center for Oral Diseases, West China Hospital of Stomatology, Sichuan University, Chengdu, China

**Keywords:** Genetics, Gene Therapy & Genetic Disease, Musculoskeletal System

## Abstract

Q. Yuan and Q. Li discuss a potential therapeutic strategy for fibrodysplasia ossificans progressiva as reported by M. Ikeya and colleagues, in this issue of *EMBO Mol Med*.

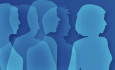

## Teaser

Fibrodysplasia ossificans progressiva (FOP) is a rare genetic disease characterized primarily by the formation of heterotopic bone in connective tissue, such as tendon, muscle, ligament and fascia (Kaplan et al, 2024). The prevalence is approximately one in 2.5 million births, with over 2,500 cases diagnosed globally. Although rare, FOP is a severe and life-limiting condition with no current cure. Due to heterotopic bone formation in various organs, patients experience intense pain, restricted mobility, difficulty with eating and speaking, hearing loss, scoliosis, and impaired pulmonary function. The average lifespan for individuals with FOP is around 40 years. Therefore, studying the pathological mechanism and developing efficient drugs for FOP are highly demanding. In this issue of *EMBO Molecular Medicine*, Zhao et al, introduce bone morphogenetic protein 9 (BMP9) as a potential therapeutic target for FOP (Zhao et al, 2024).

Heterotopic ossification (HO) initiates in response to trauma or injury to soft tissue, beginning with a painful swelling known as a flare-up. Flare-ups progress through three stages: muscle degeneration, inflammatory infiltration, and fibroproliferation, which together can last for several weeks. Following these stages, the process of endochondral bone formation often occurs at the flare-up site, where chondrocytes develop and gradually calcify into ectopic bone tissue. These flare-ups can recur, progressively debilitating patients over their lifetime (Pignolo et al, 2016).

On a molecular level, FOP is caused by a gain-of-function mutation in the activin A receptor type I (ACVR1, also known as activin-like kinase 2, ALK2), a type I receptor in the bone morphogenetic protein (BMP) pathway (Shore et al, 2006). This mutation involves a substitution of guanine with adenine, resulting in an arginine (R) to histidine (H) change at codon 206 in the intracellular glycine-serine (GS) subdomain of the ACVR1 protein, leading to constitutive activation of BMP signaling. The ACVR1^R206H^ variant is the most common, accounting for ~97% of all FOP cases. ACVR^R206H^ was shown to transmit BMP signaling independently of ligands, while also exhibiting hyperactivation in response to BMP ligands. Subsequent research revealed that ACVR^R206H^ transduces BMP signaling in response to injury-related Activin-A, a factor typically drives TGF-β signaling. Activin A is both necessary and sufficient to trigger abnormal BMP signaling, thus driving heterotopic ossification (Hatsell et al, 2015; Hino et al, 2015).

Based on the molecular understanding of FOP, various inhibitors targeting ACVR1, BMP signaling, and activin A have been developed, with some currently in clinical trials. For instance, palovarotene is the first and only FDA-approved drug for FOP patients. As a retinoic acid receptor gamma (RARγ) agonist, palovarotene inhibits BMP signaling and subsequent chondrogenesis associated with heterotopic ossification (Pignolo et al, 2022). Garetosmab, a monoclonal antibody against Activin A, demonstrated efficacy in reducing HO lesion formation and flare-ups in FOP patients during the phase 2 LUMINA-1 trial (Di Rocco et al, 2023). In addition, mTOR was identified as a mediator of Activin A-induced chondrogenesis, leading to the use of the mTOR inhibitor rapamycin, which showed anti-HO effects in FOP mouse models (Hino et al, 2018, 2017). Other drugs, including saracatinib, INCB000928, DS-6016a, and BLU-782, are designed to target ACVR1 and are in various stages of clinical development (Meng et al, 2022). However, anti-ACVR1 antibodies have been observed to promote dimerization of the ACVR1^R206H^ variant, exacerbating HO progression in FOP mouse models (Aykul et al, 2022). Importantly, current research predominantly addresses chondrogenesis and ectopic osteogenesis, which occur in the middle to late stages of FOP. The initial inflammatory flare-up in soft tissue following injury remains poorly understood. Therefore, studies are essential to elucidate the mechanisms driving the onset of HO in FOP.

In the article published in this issue of *EMBO Molecular Medicine*, Zhao and collaborators have explored the mechanism driving flare-ups in FOP. Their study identified that BMP9, secreted by macrophages, aberrantly activated TGF-β signaling in mesenchymal stem cells (MSCs) derived from FOP patient-derived induced pluripotent stem cells (FOP-iPSCs), leading to fibroproliferation and initiating flare-ups. They further showed that neutralizing BMP9 greatly reduced flare-ups and subsequent heterotopic ossification. This work highlights BMP9-mediated fibroproliferation as a critical factor in the early stages of flare-up initiation and positions BMP9 as a promising therapeutic target for managing both flare-ups and heterotopic ossification early in FOP progression.

To identify the ligand responsible for triggering abnormal fibroproliferation at the onset of FOP, Zhao et al, constructed induced-MSCs (iMSCs) obtained from FOP-iPSCs and exposed them to various TGF-β superfamily members. They found that the BMP9 specifically and markedly activated the proliferation of FOP-iMSCs. To further investigate BMP9’s effects in vivo, they constructed doxycycline (Dox)-inducible FOP (*AVCR1*^*R206H*^) mice. Injection of BMP9 into the gastrocnemius muscle of these mice led to evident flare-ups and heterotopic ossification. To explore the relationship between BMP9 and FOP further, the researchers induced muscle injury and observed elevated circulating BMP9 levels in FOP mice. Importantly, they demonstrated that BMP9 was secreted by monocytes and macrophages following injury, which in turn promoted hyperproliferation of FOP-iMSCs. Interestingly, over time, FOP-iMSCs acquired the ability to autonomously express BMP9, further contributing to heterotopic ossification.

After demonstrating that BMP9 promotes cell proliferation and heterotopic ossification (HO) in FOP, the authors investigated whether inhibiting BMP9 could mitigate HO progression. They generated FOP mice with Bmp9 knocked out and observed reduced chondrocyte formation and slowed HO progression. In addition, subcutaneous injection of a BMP9-neutralizing antibody significantly decreased cell proliferation and alleviated early HO development. Notably, they found that early administration of the BMP9 antibody shortly after injury, rather than delayed intervention, effectively mitigated FOP lesion formation. These findings underscore the critical role of BMP9 in the early stages of FOP progression.

Finally, the authors identified BMP9 aberrantly activated Smad2/3-mediated TGF-β signaling in FOP MSCs, rather than its canonical activation of Smad1/5/9-mediated BMP signaling. This atypical signaling is mediated by the ACVR1 type I receptor and additionally requires the involvement of ACVR2A and BMPR2 type II receptors.

In summary, Zhao and collaborators focused on the flare-up stage of FOP lesions, revealing for the first time the critical role of BMP9 in driving abnormal MSC proliferation and heterotopic ossification following injury in FOP mice. This study introduces BMP9 as a potential therapeutic target for FOP and suggests that early intervention during the flare-up stage may effectively alleviate disease progression. Furthermore, additional research is needed to identify other ligands that contribute to the initiation of FOP and drive abnormal MSC proliferation and heterotopic ossification.
